# Case of Cyanosis—Opening between the Ventricles, near the Mouth of the Aorta, with Contraction of the Pulmonary Artery

**Published:** 1849-07

**Authors:** C. A. Lee


ART. II.—*Case of Cyanosis—Opening between the Ventricles, near the Mouth of the Aorta, with Contraction of the Pulmonary Artery.* By CHARLES A. LEE, M. D., etc.

T. R., a lad aged 15, had been affected since birth with symptoms of an open *foramen ovale*, or some communication between the right and left chambers of the heart. The skin had been constantly of a deep cerulean hue from birth; he had been subject to frequent attacks of dyspnea on very slight exertion; great weakness of the voluntary muscles, so that when seized with an asthmatic attack, from mental excitement, or too sudden exercise, he would turn black in the face, and fall suddenly to the ground; in a few minutes the attack would pass off, and he would cautiously rise, and slowly walk away. During these paroxysms, his consciousness did not appear to be lost; nor would there be any convulsive motions. There was, also, great debility of the nervous system; and though naturally amiable and affectionate in his disposition, his mental faculties were weak; his memory being very imperfect, so that he could not remember events which happened from day to day. He was wayward and eccentric, and sought out the lowest companions; would often wander away from home; and has been known to return, after an absence of some days or weeks, without any of the clothes on with which he left home, but clad in an old shirt and coarse pantaloons, saying that he had given away his clothes. He had but little if any regard for truth; indeed his moral perceptions were altogether obtuse; nor could he be taught to shun falsehood, or have any just conceptions of its nature, or the obligations of moral and and filial duty. There was no want of development of any part of the body; he being as large as boys usually are at his age. The conformation of the head, also, was not defective, there being nothing remarkable either as to its size or shape. The temperature of the surface was ordinarily, and probably uniformly, below the standard of health.

In connection with these general phenomena there were some local symptoms which aided in the diagnosis. Auscultation revealed a strong murmur, which appeared almost superficial, and whose maximum was about on a level with the lower border of the third rib. This murmur was heard more distinctly along the course of the aorta than along that of the pulmonary artery. There was increased dullness on percussion, over a larger space than natural, indicating hypertrophy or dilatation of the ventricles. The phenomena clearly pointed to a communication between the right and left cavities of the heart, accompanied with an enlargement of the organ. Early in May, he was attacked with scarlet fever, accompanied with meningitis, and died after an illness of about three weeks. During his illness, the color of the surface was most of the time restored, so as to present the natural hue of the skin; towards the close of his illness, there were, however, paroxysms, during which the leaden hue became strongly marked.

*Autopsy*—12 hours after death.

Emaciation very great—nothing remarkable in the color and external appearance of the body.

*Head.*—On raising the cranium, the dura mater was found strongly adherent to the skull, and extremely injected. There was slight effusion beneath the arachnoid; but the whole surface of the brain exhibited marks of the highest degree of congestion; all the vessels and sinuses being turgid with blood. The lateral ventricles were distended with serum, and the plexus choroides fully injected. The substance of the brain was preternaturally firm, resembling almost a brain some time soaked in alcohol. This phenomenon was doubtless the result of the disease of which he died.

*Lungs.*—Nothing remarkable was observed in connection with the lungs; they containing about the usual quantity of blood.

*Heart.*—On opening the pericardium, a small quantity of serum was found in its cavity. The heart was evidently considerably enlarged throughout. The pulmonary artery in this malformation is generally very narrow, and its sigmoid valves either partially or wholly wanting,

On opening the cavities, *a free communication was discovered between the two ventricles, at the very origin of the aorta, at least one inch in diameter;* the septum for this distance being entirely wanting; thus affording a free passage of blood between the two sides of the heart, and from each ventricle into the upper part of the aorta. The upper part of the septum was of a firm, tendinous structure. The mouth of the pulmonary artery was in a great measure closed, by a firm membrane reflected from its inner coat; so that the calibre of the vessel at its narrowest part, was not more than two lines in diameter at about one inch from its origin. Its semilunar valves were wanting. Between this and the heart the pulmonary artery was of the usual size, and, like the aorta, communicated freely with the left, by an oblique, duct-like passage. The mouth of the aorta was also somewhat contracted. The sigmoid valves, as well as the mitral and Eustachian, were healthy. The walls of the ventricles were thickened and dilated; affording a very good illustration of hypertrophy, with dilatation. No other morbid appearances were discovered about the heart, except the presence of several firm, flesh colored, semi-organized polypi, or masses of fibrine, in the cavities—one in the left ventricle passing far up into the aorta. Some of these, it was thought, had been formed some days previous to death.

*Liver.*—On the under surface of the liver, was attached a cyst of the size of a hen's egg, filled with a yellow, serous fluid, abounding in cholesterine. Its substance was otherwise healthy.

Nothing remarkable was observed in the other organs.

*Remarks.*—The above malformation, so far as I am acquainted, has not hitherto been regarded. There are instances where the aorta has arisen from both ventricles, the septum of the latter being wanting at the valves of the aorta; and in connection with this, contraction of the pulmonary artery, and an open *foramen ovale.* Cases, too, are on record, where the pulmonary artery has arisen from both ventricles, the foramen ovale remaining patent; the aorta being entirely supplied by this artery; the aorta pursuing its usual course. Meckel remarks, (*Manual of Anatomy,* Am. Ed. Vol. II. p. 220,) "The septum of the ventricles is perforated generally in one determinate place, viz. the base; so that sometimes the aorta, but more unfrequently the pulmonary artery, arises from both ventricles; *in the latter case the aorta arises as usual,* but forms only an ascending portion, and terminates in the left subclavian artery, and the ascending aorta comes entirely from the pulmonary artery." A communication between the ventricles, through an opening in their septum, with closing of foramen ovale, has been met with, though rarely, by pathologists. Five cases of this kind have been collected by Louis;* one by Jackson, of the English General Whipple, and another is related by Otto †—a deficiency of the ventricular septum with a patulous state of the foramen ovale, is more

* Mem. Anatomico-pathol. p. 301.

† Tettene Beobachtungen, p. 96.

common. See Richerand's Physiology, (Am. Ed.) for a very interesting case in a man aged 40. Hasse (Leichenoffnungen, p. 162) has also recorded an interesting case of this kind in a girl, aged 19. The opening of the ventricles, in nearly every case, has been found near the base of the heart, immediately below the origin of the pulmonary artery and aorta. The width of the ventricular opening has varied from two lines to an inch in diameter; and in one case, (recorded by Burdach, in a boy of 16 years,) there was almost an entire deficiency of the ventricular septum. Farre has published two cases, where the pulmonary artery arose from both ventricles, (one aged 3 weeks, the other 8 months.) The origin of the aorta from both ventricles is not so uncommon. In some instances, life has been long protracted under these circumstances, as in some cases recorded by Louis, Albers, Bouilland, and Meyer. Cerutti has published a case of this kind in a girl of 9 years; Sandifort, a boy of 13; Albers, a girl of 17; Tomasini, a woman of 25; Meyer, a girl of 17.

The bearings of the case which I have briefly described, are not uninteresting in a physiological and pathological point of view. We have seen that the mental and bodily functions were all imperfectly performed; that the moral, intellectual, and physical powers languished; and that the phenomena of life were closely allied to those of the reptile tribe—as the malformation itself arises from an arrest, at an early period, of cardiac development, giving rise to a formation analogous to that which we find in the hearts of some of the lower classes of animals, particularly the crustacea, the mollusca, and the reptiles. From the small opening in the pulmonary artery, and the removal of so large a portion of the ventricular septum, it is evident that the venous blood was freely sent into the left ventricle and the aorta, without going through the lungs; indeed, the calibre of the pulmonary artery would not have admitted the sixth part of the usual quantity of the blood, to have passed through it. *Cyanosis* was necessarily a strongly marked symptom in the case. To what extent were the morbid phenomena in this case owing to the contraction of the pulmonary artery, or the right arterial orifice, thus preventing hematosis, if not the free egress of blood from the right ventricle? As the dilatation and thickening of the cardiac walls were not, however, very great, and the ventricular septum largely deficient, it may be doubted whether the cyanosed state of the skin could be imputed, wholly, to "venous congestion," as advocated by Dr. Stille and others, as the characteristic phenomena in cyanosis. It is admitted, that the blue disease does not necessarily follow from an open state of the foramen ovale; for Cloquet and Louis have clearly demonstrated, that the mingling of the black and red blood does not take place through an orifice in the septa of the heart, when the walls of the two cavities which communicate with each other, are of equal thickness and strength, and even where their strength is unequal, provided the heart's orifices are sufficiently ample. In the present case, however, owing to the contraction of the pulmonary artery, the venous blood flowed freely through the auricular septum into the left ventricle, and thus through the systemic circulation. The cyanosed condition, in this case, would seem to have been, in some degree, at least, occasioned by the dark condition of the blood, as well as by the venous obstruction. In an open state of the foramen ovale, we know that the discoloration is not permament throughout life; that the cyanotic phenomena occur periodically only, and sometimes at long intervals; are brought on by sudden exercise, mental emotions, &c., and then disappear after a short time. Now, it is said, that if the blueness depended on the character of the blood, these alternations would not happen. But is it not probable, that under ordinary circumstances, the two kinds of blood are not mixed in the heart, and that this only occurs during these paroxysms, when so much blood is forced into the right side of the heart as to cause a current of venous blood through the opening into the left ventricle?

Rokitansky, however, maintains* that even a deficiency in the ventricular septum, does not produce cyanosis, without some co-existing anomaly of the arterial trunks; that the less these deviate from their normal condition, the more trifling and transient the cyanotic phenomena will be, and will only occur under certain circumstances, such as mental emotions, bodily exertion, and diseases of the lungs. Where there is an almost total occlusion of the mouth of the pulmonary artery, and the aorta takes its rise, as in the above instance, from both ventricles, Rokitansky maintains that the aorta is insufficient to carry off the blood of both ventricles, and that cyanosis must result from the impeded entrance of the venous blood into them. The same result would also happen from the contraction of the cavity of a ventricle; it being insufficient for the whole mass of blood, venous congestion results, and cyanosis follows.

For an opportunity of witnessing the above case, and the post-mortem appearances, I am indebted to the politeness of my friend Sumner Rhoades, M. D., of Geneva.

---

* Buffalo Medical Journal for May, p. 752.

